# Proliferating Cell Nuclear Antigen Binds DNA Polymerase-β and Mediates 1-Methyl-4-Phenylpyridinium-Induced Neuronal Death

**DOI:** 10.1371/journal.pone.0106669

**Published:** 2014-09-03

**Authors:** Zhentao Zhang, Zhaohui Zhang, Hongcai Wang, Guoxin Zhang, Dan Hu, Jing Xiong, Nian Xiong, Tao Wang, Xuebing Cao, Ling Mao

**Affiliations:** 1 Department of Neurology, Renmin Hospital of Wuhan University, Wuhan, China; 2 Department of Neurology, Union Hospital, Tongji Medical College, Huazhong University of Science and Technology, Wuhan, China; Institute of Molecular Genetics IMG-CNR, Italy

## Abstract

The mechanisms leading to dopaminergic neuronal loss in the substantia nigra of patients with Parkinson disease (PD) remain poorly understood. We recently reported that aberrant DNA replication mediated by DNA polymerase-β (DNA pol-β) plays a causal role in the death of postmitotic neurons in an *in vitro* model of PD. In the present study, we show that both proliferating cell nuclear antigen (PCNA) and DNA pol-β are required for MPP^+^-induced neuronal death. PCNA binds to the catalytic domain of DNA pol-β in MPP^+^-treated neurons and in post-mortem brain tissues of PD patients. The PCNA-DNA pol-β complex is loaded into DNA replication forks and mediates DNA replication in postmitotic neurons. The aberrant DNA replication mediated by the PCNA-DNA pol-β complex induces p53*-*dependent neuronal cell death. Our results indicate that the interaction of PCNA and DNA pol-β contributes to neuronal death in PD.

## Introduction

Parkinson disease (PD) is one of the most common neurodegenerative diseases in the elderly. It is characterized by the progressive degeneration of dopaminergic neurons in the substantia nigra pars compacta. The molecular mechanisms leading to neuronal death in PD have not been fully elucidated. It has been reported that some of the degenerating postmitotic neurons in PD patients re-activated the molecular program that normally guides proliferating cells through the cell cycle to mitotic division [1], [2], [3]. It is well-recognized that mature neurons are terminally differentiated and unable to re-enter the cell cycle. However, the ectopic expression of cell cycle-related proteins was reported in vulnerable neurons of post-mortem specimens from PD and Alzheimer disease (AD) patients [4], [5], [6]. Furthermore, some of the vulnerable neurons from PD and AD patients are polyploid, indicating some of the neurons have finished at least part of the DNA replication [1], [7], [8]. DNA replication precedes neuronal death during the onset and progression of AD [9]. On the other hand, inhibition of the DNA replication in postmitotic neurons attenuates neuronal death under several pathogenic conditions, indicating that aberrant DNA replication might be a common pathway underlying neuronal death in neurodegenerative diseases such as PD and AD [10], [11], [12], [13]. However, the link between DNA replication and neuronal death is still missing.

We and others showed that the aberrant DNA replication in PD and AD models is not mediated by the canonical replicative DNA polymerase-ε (pol-ε) or DNA pol-δ, but by DNA pol-β [10], [14], [15]. The primary function of DNA pol-β is associated with base excision repair and other ways of DNA repair [16]. We found that DNA replication mediated by DNA pol-β is required for neuronal death [10]. However, the exact molecular mechanisms through which DNA pol-β mediates DNA replication and neuronal death are not clear. Usually, DNA polymerases interact with other proteins to exert their functions. Proliferating cell nuclear antigen (PCNA) is an evolutionarily conserved protein. Its major function is to act as a processivity factor for DNA pol-δ during DNA replication. It is also associated with other vital cellular processes such as cell cycle control, DNA repair, and chromatin remodeling [17]. PCNA was found to be expressed in the degenerating neurons from PD patients but not in the healthy neurons from normal controls [1].

In the present study, we used an *in vitro* model of PD and human post-mortem tissue to examine the interaction of PCNA and DNA pol-β in the pathogenesis of PD. We found that PCNA binds to the catalytic domain of DNA pol-β both *in vitro* and *in vivo*. The interaction of PCNA and DNA pol-β is required for the aberrant DNA synthesis and neuronal death.

## Materials and Methods

### Primary neuron culture

Cerebral cortical cultures were prepared from 18-day mouse embryos as previously described [14]. This study was carried out in strict accordance with the recommendation in the *Guide for the Care and Use of Laboratory Animals* of the National Institutes of Health and local laws. All procedures were approved by Ethics Committee of Wuhan University. All efforts were made to minimize animal stress and suffering. The culture were maintained in basal medium with Eagle’s salts (BME) supplemented with 10% fetal calf serum (FCS), 2 mM l-glutamine, 100 µg/ml streptomycin, 100 U/ml penicillin, and 25 mM KCl. 10 µM cytosine arabinoside was added to the medium 24 h after plating and maintained for 24 h to prevent glial proliferation. The neurons were subsequently maintained in serum-free neurobasal medium (Invitrogen, Carlsbad, CA) containing 2% B27 supplement and 2 mM glutamine at 37°C in a humidified 5% CO_2_ incubator. All the experiments were conducted after 10 days of *in vitro* culture.

### Hoechst staining

The cells were fixed in 10% formaldehyde for 10 min at room temperature. After washing with PBS, the cells were incubated with 5 µg/ml Hoechst 33258 (Sigma, St. Louis, MO) for 10 min. The stained nuclei were visualized by fluorescence microscopy. Cells with condensed chromatin and/or fragmented nuclei were considered apoptotic. The number of apoptotic cells was determined by counting at least 500 cells from each sample over four separate experiments.

### TUNEL staining of apoptotic cells

Neuronal apoptosis was detected with the *in situ* cell death detection kit (Indianapolis, IN) as previously reported [18]. Briefly, the slides were incubated overnight at 4°C with anti-MAP2 antibody (Sigma). After being washed with TBS, the sections were incubated with Alexa Fluor 488-coupled secondary antibodies. The sections were then incubated with TUNEL reagent for 1 h at room temperature. After a PBS wash, images were acquired through an AxioCam camera on an Axiovert 200 M microscope (Zeiss). The apoptotic index was expressed as the percentage of TUNEL-positive neurons out of the total number of MAP2-positive neurons.

### Overexperssion and knockdown of PCNA, DNA pol-β, and P53

Human cDNAs for PCNA and DNA pol-β were cloned into the retroviral vector pBabe-hygro. Point mutation was introduced using QuikChange Site-Directed Mutagenesis Kit. Retroviruses were produced in 293FT cells using the packaging plasmids pHDM-G and pMD.MLVogp. The shRNA lentiviral particles were bought from Santa Cruz Biotechnology (Santa cruz, CA).

### GST pull-down assay

HEK293 cells were maintained in DMEM, supplemented with 10% fetal bovine serum (FBS), 2 mg/ml glutamine and 100 units penicillin-streptomycin at 37°C with 5% CO_2_ atmosphere in a humidified incubator. We used plasmids expressing the N-terminal GST-tagged proteins: GST-DNA pol-β full length, GST-DNA pol-β DNA binding domain, and GST-DNA pol-β catalytic domain. Ten-cm dishes of HEK293 cells were transfected with 10 µg DNA by the calcium phosphate precipitation method. 48 hours after transfection, the cells were collected, lysed in lysis buffer, centrifuged for 15 min at 16,000 g. The supernatant was incubated with Glutathione Sepharose 4B beads for 4 hours at 4°C. After extensive washing, the bound proteins were eluted from the beads by boiling in Laemmli sample buffer and subjected to Western blot analyses.

### BrdU incorporation assay

BrdU (Sigma) was added to neuronal cultures at a final concentration of 10 µM. The cells were incubated for 4 h and then fixed in 10% formaldehyde for 10 min at room temperature. The DNA was denatured by incubation with 2 N HCl for 30 min at room temperature, followed by neutralization with 0.1 M borate buffer, pH 8.5. The incorporation of BrdU into the nucleus was assayed by immunofluorescence using an anti-BrdU monoclonal antibody (1∶100; Becton Dickinson, Franklin Lakes, NJ), followed by counterstaining of the cells with Hoechst 33258. The number of BrdU-positive nuclei were divided by the total number of Hoechst-stained nuclei and expressed as a percentage of the total number of nuclei.

### Western blot analysis

The neurons was lysed in lysis buffer (50 mM Tris, pH 7.4, 40 mM NaCl, 1 mM EDTA, 0.5% Triton X-100, 1.5 mM Na3VO4, 50 mM NaF, 10 mM sodium pyrophosphate, 10 mM so­dium β-glycerophosphate, supplemented with protease inhibitors cocktail), and centri­fuged for 15 min at 16 000 g. The supernatant was boiled in SDS loading buffer. After SDS-PAGE, the samples were transferred to a nitrocellulose mem­brane. Western blotting analysis was performed with a variety of antibodies. The anti-DNA pol-β, anti-PCNA, anti-β tubulin, anti-ki67, and mouse anti-p49 antibodies were purchased form Santa Cruz Biotechnology. The anti-active caspase-3, and anti-p53 antibodies were purchased from Cell Signaling Technology (Boston, MA). Anti-cdc45 and rabbit anti-p49 antibodies were purchased from Abcam (Cambridge, UK).

### Human tissue samples

Post-mortem brain samples were dissected from frozen brains of 4 PD cases and 4 age-matched normal controls. PD was diagnosed according to the UK Parkinson's disease society brain bank criteria. This present study was approved by the ethics committee at Wuhan University. The human tissue samples were from Chinese Brain Bank Center (Wuhan). Permission was requested from family members, and permission was granted to use post-mortem brain tissue samples from PD patients.

### Preparation of DNA/protein fragments

The DNA/protein fragments were prepared as previously described [14]. Briefly, the MPP^+^-treated neurons were crosslinked with 1% formaldehyde for 10 min at room temperature (RT), followed by a quenching of the cross-linking reaction with 125 mM glycine for 5 min. The cells were harvested, and resuspended in three packet cell volumes (pcv) of RSB buffer [10 mM Tris-HCl, 10 mM NaCl, 3 mM MgCl2, pH 8.0] and homogenized by 25 strokes in a Dounce homogenizer. Nuclei were collected by centrifugation at 750 g for 10 min, and were then resuspended in buffer E [10 mM Tris-HCl, 10 mM Na2S2O5, 1 mM EDTA-KOH plus 1 M NaCl, 0.1% NP-40, and protease inhibitor cocktail diluted 1∶100] to get rid of the unbound proteins. Cross-linked nuclear proteins were collected by centrifugation at 750 g for 10 min, and then resuspended in 0.5 pcv lysis buffer (50 mM Tris/HCl, 10 mM EDTA, 1% SDS, and protease inhibitor cocktail) and maintained at 4°C for 10 min. Then the DNA/protein complex was sheared by sonication for 3 min with output control setting 5. Debris was cleared by centrifugation at 10,000 g for 10 min at 4°C.

### Co-immunoprecipitation

The cultured cells or human brain tissue samples were lysed in lysis buffer and centri­fuged for 15 min at 16,000 g. The supernatant was incubated with anti-PCNA antibody or anti-DNA pol-β antibody and protein A/G-agarose overnight at 4°C. After extensive washing, the bound proteins were eluted from the beads by boiling in Laemmli sample buffer and subjected to Western blot analyses.

### Statistical analysis

All of the data were presented as mean ± SEM. Statistical analysis was performed using either Student’s t-test (two-group comparison) or one-way ANOVA followed by the LSD post-hoc multiple comparison test (more than two groups). The level of significance was set for p-value <0.05.

## Results

### Both PCNA and DNA pol-β are required for neuronal death induced by MPP^+^

**Figure 1:**
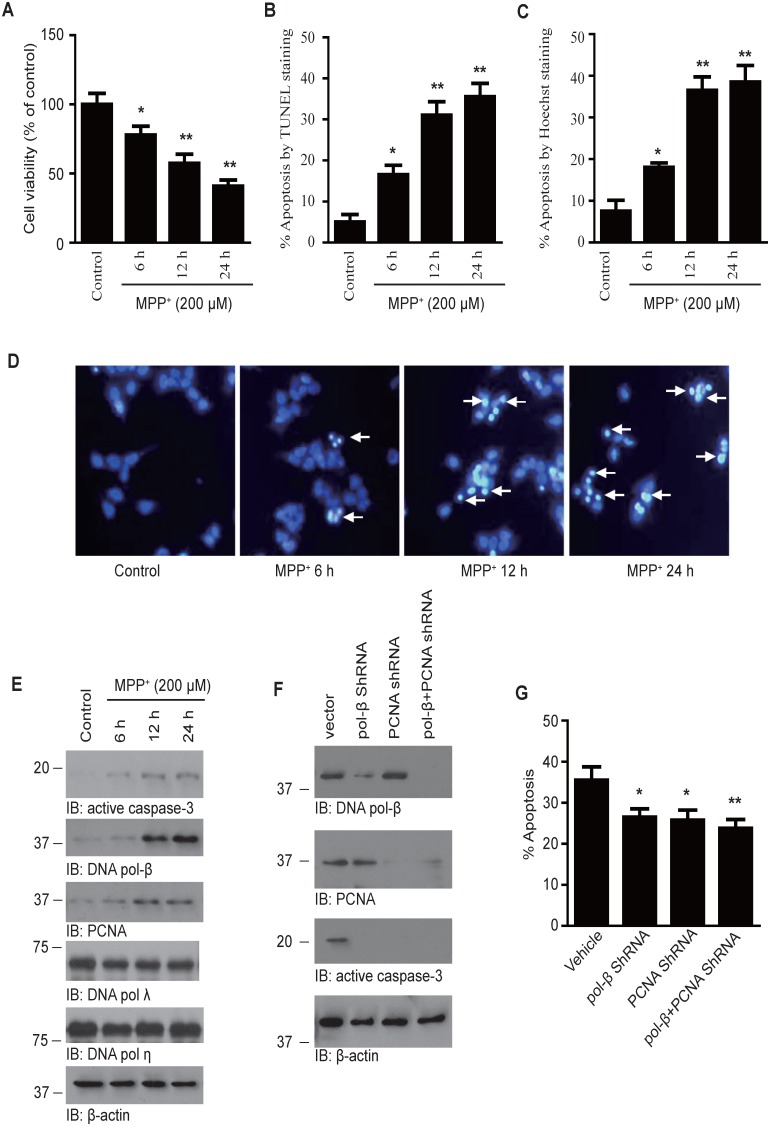
PCNA and DNA pol-β are required for neuronal death induced by MPP^+^. (**A**) Cell viability assay. The neurons were exposed to 200 µM MPP^+^ for 6 h, 12 h, and 24 h, respectively. Cell viability was determined using the MTT method. Data are expressed as a percentage of the value in untreated control cells. (**B**) Apoptosis rata determined by TUNEL staining. After the neurons were exposed to 200 µM MPP^+^ for 6 h, 12 h, or 24 h, they were immunostained with neuronal marker MAP2. Neuronal apoptosis was detected by TUNEL staining. (**C**) Apoptotic rate determined by Hoechst staining. (**D**) Representative images of cells after Hoechst staining. Arrows indicate the apoptotic cells. (**E**) Immunoblotting analysis of the expression of PCNA, DNA pol-β, DNA pol-λ, DNA pol-η, and active caspase-3 after treatment with MPP^+^. The protein levels of PCNA, DNA pol-β and active caspase-3 increased significantly after the neurons were exposed to 200 µM MPP^+^ for 12 h or 24 h. (**F**) Immunoblotting experiments showing the efficiency of PCNA and DNA pol-β shRNA. The neurons were infected with PCNA and/or DNA pol-β shRNA lentivirus for 24 h, and then exposed to 200 µM MPP^+^ for 12 h. The expression of PCNA and DNA pol-β was knocked down by their specific shRNAs. (**G**) Effect of PCNA and DNA pol-β knockdown on neuronal apoptosis was detected by TUNEL staining. Both shRNAs attenuated neuronal apoptosis induced by MPP^+^. Date represent the mean ± SEM from four independent experiments. *p<0.05, **p<0.01 compared with control group.

We first determined the toxic effect of MPP^+^ on cultured cortical neurons. MTT assay showed that cell viability decreased to 77.55±13.00%, 57.25±13.30%, and 40.75±9.03% after the neurons were incubated with 200 µM MPP^+^ for 6 h, 12 h, and 24 h, respectively (Figure 1A). To further determine neuronal apoptosis induced by MPP^+^, we immunostained the slides with the neuronal marker MAP2 and then stained with TUNEL *in situ* cell death detection Kit. MPP^+^ treatment provoked neuronal apoptosis in a time-dependent manner. The apoptotic rate increased from 5.00±3.74% to 31.75±6.65 after 12 h of treatment with 200 µM MPP^+^ (Figure 1B). Neuronal apoptosis was confirmed by Hoechst staining. Condensed and/or fragmented nuclei were found after exposure to MPP^+^ (Figure 1C, D). Furthermore, the protein level of active caspase-3 was also increased in MPP^+^-treated neurons (Figure 1E, upper panel).

Since we and others found that neuronal death in PD is associated with aberrantly activated cell cycle events, and DNA pol-β may play a role in neuronal death [10], [15], we tested the protein levels of DNA polymerases and the DNA polymerase cofactor PCNA. The endogenous expression of DNA pol-β and PCNA was very low. However, the expression of both proteins increased significantly after the neurons were treated with 200 µM MPP^+^ for 12 h to 24 h. The expression of DNA pol λ and DNA pol η was not affected by MPP^+^ (Figure 1E). To further investigate the role of increased DNA pol-β and PCNA in neuronal death, we knocked down the expression of both proteins by infecting the cells with their specific shRNA lentivirus, and treated the neurons with 200 µM MPP^+^ for 12 h (Figure 1F). We found that knockdown of PCNA or DNA pol-β attenuated MPP^+^-induced neuronal death as detected by TUNEL staining. Knockdown of PCNA or DNA pol-β also attenuated the expression of active caspase-3 (Figure 1F, G). These results demonstrate that both DNA pol-β and PCNA are required for neuronal death induced by MPP^+^.

### PCNA binds to the catalytic domain of DNA pol-β

**Figure 2:**
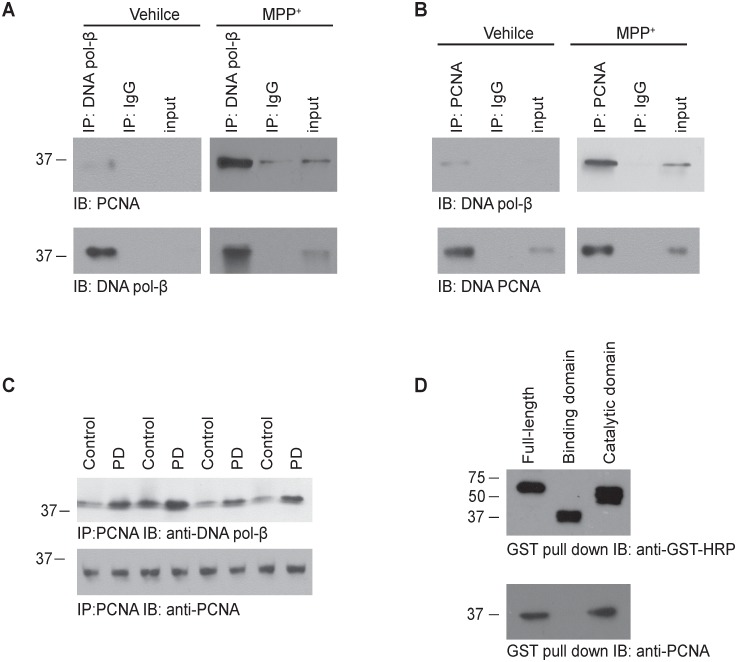
PCNA binds DNA pol-β in MPP^+^-treated neurons and in post-mortem brain tissue of PD patients. (**A–B**) Coimmunoprecipitation of PCNA and DNA pol-β in vehicle- and MPP^+^-treated neurons. Coimmunoprecipitation studies were carried out with lysates prepared from neurons treated with vehicle (A) or 200 µM MPP^+^ for 12 h (B). The lysates were immunoprecipitated with an anti-DNA pol-β antibody and then immunoblotted using an anti-PCNA antibody, or immunoprecipitated with an anti-PCNA antibody and then immunoblotted using an anti-DNA pol-β antibody. Mouse IgG was used as a negative control. (**C**) Coimmunoprecipitation of PCNA and DNA pol-β in post-mortem brain tissue of PD patients. Human brain lysates from PD patients and control were immunoprecipitated with an anti-PCNA antibody and then immunoblotted using anti-DNA pol-β and anti-PCNA antibody. (**D**) GST pull-down assay. The HEK293 cells were transfected with plasmids expressing GST-DNA pol-β full length, GST-DNA pol-β DNA binding domain, and GST-DNA pol-β catalytic domain, respectively. 48 h after transfection, GST-tagged proteins were pulled down with Glutathione Sepharose 4B beads, and then immunoblotted using an anti-PCNA antibody. PCNA binds to the catalytic domain but not the DNA binding domain of DNA pol-β.

In order to test whether PCNA directly interacts with DNA pol-β, we treated the neurons with 200 µM MPP^+^ for 12 hours, and carried out co-immunoprecipitation experiments. Interestingly, we found that DNA pol-β was reciprocally co-immunoprecipitated with PCNA in the MPP^+^-treated neurons but not in the control neurons (Figure 2A, B). To further test whether the interaction of PCNA and DNA-pol-β also happens in the brain of PD patients, we did co-immunoprecipitation experiments with brain tissues from PD patients and healthy controls. As shown in Figure 2C, we found that DNA pol-β was extensively co-immunoprecipitated with PCNA in tissues from PD patients, and to a lesser extent in tissues from age-matched controls, suggesting the interaction between PCNA and DNA pol-β exits in normal brain tissues, but this interaction is enhanced during the onset and progress of PD.

DNA pol-β contains two tightly folded domains: the DNA binding domain (8 kDa) and the catalytic domain (31 kDa). To test which domain is required for its binding to PCNA, we made three recombinant plasmid constructs expressing GST-DNA pol-β full length, GST-DNA pol-β DNA binding domain, and GST-DNA pol-β catalytic domain, respectively, and transfected these plasmids into HEK293 cells. 48 h after transfection, GST pull-down assay showed that PCNA was pulled down by DNA pol-β full length and its catalytic domain, but not by its DNA binding domain. Our results indicate that the catalytic domain of DNA pol-β is required for its interaction with PCNA (Figure 2D). This result is in consistent with the previous report that the PCNA interacting motif (PIM)-like sequence of DNA pol-β mediates its interaction with PCNA [19].

### PCNA and DNA pol-β are loaded into DNA replication forks in postmitotic neurons challenged with MPP^+^

**Figure 3:**
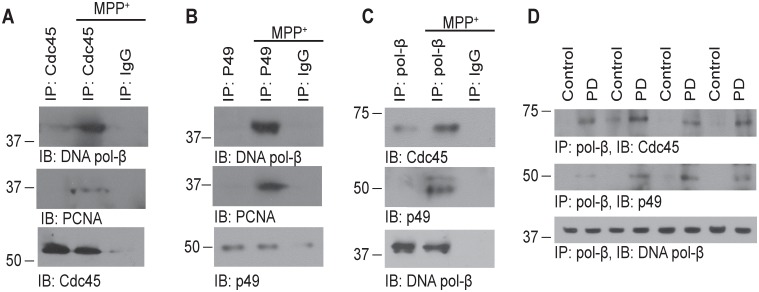
Loading of PCNA and DNA pol-β into DNA replication forks. (**A**) The neurons were treated with 200 µM MPP^+^ or vehicle for 12 h. The nucleoprotein fragments were immunoprecipitated using an anti-Cdc45 antibody and immunoblotted using anti-DNA pol-β and anti-Cdc 45 antibodies. (**B**) The nucleoprotein fragments were immunoprecipitated using a mouse anti-p49 antibody and immunoblotted using anti-DNA pol-β and rabbit anti-p49 antibodies. (**C**) The neurons were treated with 200 µM MPP^+^ or vehicle for 12 h. The nucleoprotein fragments were immunoprecipitated using anti-DNA pol-β antibody and immunoblotted using anti-Cdc45, anti-p49, and anti-DNA pol-β antibodies. (**D**) The nucleoprotein fragments of PD brain tissue or control brain tissue were immunoprecipitated using anti-DNA pol-β antibody and immunoblotted using anti-Cdc45, anti-p49, and anti-DNA pol-β antibodies.

To explore whether the binding of PCNA and DNA pol-β mediates aberrant DNA replication induced by MPP^+^, we tested the loading of the PCNA-DNA pol-β complex into the DNA replication forks. Cdc45 is a marker for typical DNA replication forks [20]. Co-immunoprecipitation experiments found that Cdc45 co-immunoprecipitated with PCNA and DNA pol-β after the neurons were exposed to MPP^+^ for 12 h (Figure 3A). To confirm this result, we used another marker of DNA replication forks, the p49 subunit of DNA primase, which initiates DNA replication [21]. P49 subunit also co-immunoprecipitated with PCNA and DNA pol-β (Figure 3B). These results was confirmed by reciprocal immunoprecipitation assay (Figure 3C), indicating that The PCNA-DNA pol-β complex is loaded into DNA replication forks in postmitotic neurons challenged with MPP^+^. We also tested the loading of PCNA and DNA pol-β into DNA replication forks in post-mortem brain tissues. Both Cdc45 and P49 subunit were coimmunoprecipitated with DNA pol-β in PD brain tissues but not in control tissues (Figure 3D).

### The interaction of PCNA and DNA pol-β is required for aberrant neuronal DNA replication

**Figure 4:**
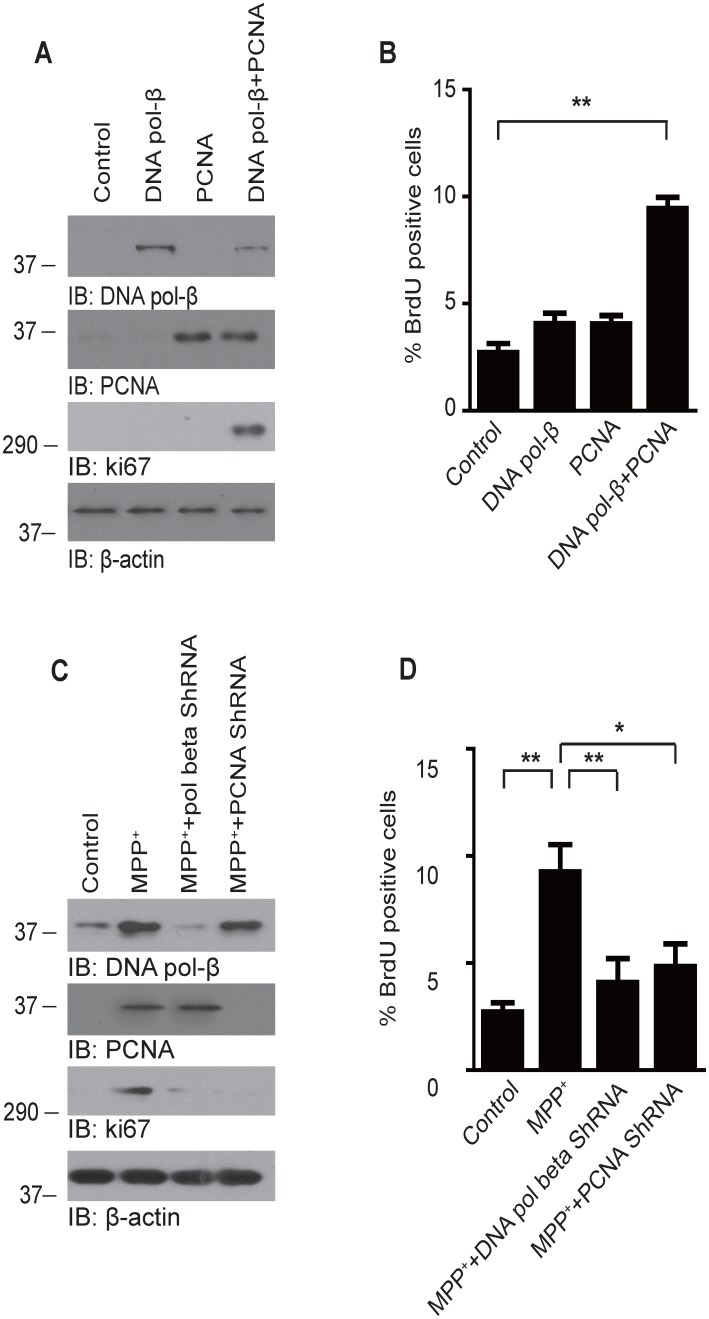
The interaction of PCNA and DNA pol-β is required for neuronal DNA replication. (**A**) Expression of DNA pol-β, PCNA and cell proliferation marker ki67 in neurons infected with DNA pol-β-expressing retroviruses, PCNA-expressing retroviruses, or both. (**B**) DNA replication in neurons expressing DNA pol-β and/or PCNA. 24 hours after infection, the neurons were incubated with 10 µM BrdU for 4 h, followed by immunostaining with anti-BrdU antibody. The percentage of BrdU positive nuclei increased only when the neurons express PCNA and DNA pol-β at the same time. (**C**) Expression of DNA pol-β, PCNA and ki67 in neurons infected with PCNA and DNA pol-β specific shRNA lentivirus. (**D**) The neurons were exposed to 200 uM MPP^+^ in the presence or absence of PCNA and DNA pol-β specific shRNA lentivirus. The percentage of cells with DNA replication was determined by BrdU incorporation assay. Date represent the mean ± SEM from four independent experiments. *p<0.05, **p<0.01.

To determine whether the PCNA-DNA pol-β complex mediates the aberrant neuronal DNA replication, we overexpressed PCNA and DNA pol-β in cultured neurons using retroviral vectors, and tested whether the ectopic expression of PCNA and DNA pol-β can initiate DNA replication. We found that neither PCNA nor DNA pol-β was sufficient to initiate DNA replication. However, when the two proteins were overexpressed at the same time, the cells initiated DNA replication even in the absence of MPP^+^ (Figure 4A, B). On the other hand, knockdown of DNA pol-β or PCNA inhibited aberrant cell cycle re-entry induced by MPP^+^ (Figure 4C, D). To confirm that BrdU incorporation indeed represents cell proliferation but not DNA synthesis that is not related to cell proliferation, such as normal DNA turnover and DNA repair, we tested the expression of Ki67, a well characterized marker for cell proliferation [22]. The level of ki67 increased significantly in neurons overexpressing both DNA pol-β and PCNA, and decreased by knocking down DNA pol-β or PCNA. These results indicate that the PCNA-DNA pol-β complex is required for aberrant neuronal DNA replication (Figure 4A, C).

### The interaction of PCNA and DNA pol-β induces p53-dependent neuronal apoptosis

**Figure 5:**
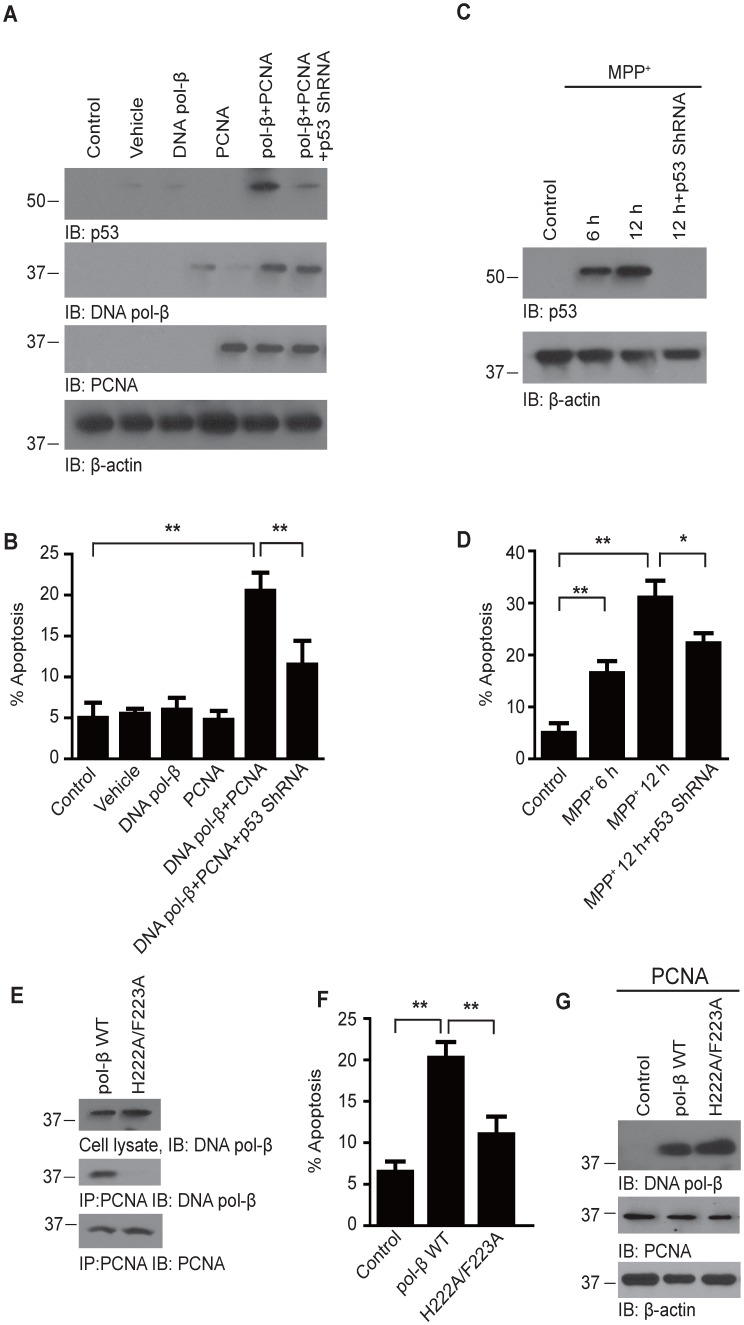
The interaction of PCNA and DNA pol-β induces p53-dependent neuronal apoptosis. (**A**) The expression of p53 in neurons infected with retroviruses expressing DNA pol-β, PCNA in the presence or absence of p53 shRNA lentiviral particles. Overexpression of DNA pol-β and PCNA increased the protein level of p53, which was abolished by p53 shRNA lentivirus. (**B**) Apoptotic rate determined by Hoechst staining. The apoptotic rate increased significantly when the neurons overexpress DNA pol-β and PCNA at the same time. Knockdown of p53 attenuated neuronal apoptosis induced by DNA pol-β and PCNA. (**C**) Immunoblot analysis of p53 in MPP^+^-treated neurons. The protein level of p53 increased after the neurons were incubated with MPP^+^. P53 shRNA lentivirus effectively knocked down the expression of p53. (**D**) Effect of p53 knockdown on neuronal apoptosis. P53 shRNA significantly attenuated neuronal death induced by MPP^+^. Date represent the mean ± SEM from four independent experiments. *p<0.05, **p<0.01. (**E**) The interaction of PCNA and DNA pol-β was abolished by H222A/F223A mutation. Neurons were infected with retrovirus encoding PCNA together with retrovirus encoding wild-type DNA pol-β or H222A/F223A mutant DNA pol-β. The lysates were immunoprecipitated with an anti-PCNA antibody and then immunoblotted using an anti-DNA pol-β antibody. (**F**) H222A/F223A mutant attenuate the toxic effect of DNA pol-β. The neurons were infected with retrovirus encoding PCNA together with retrovirus encoding wild-type DNA pol-β or H222A/F223A mutant DNA pol-β. Neuronal apoptosis was assessed by TUNEL staining. (**G**) Western blot analysis of PCNA and DNA pol-β expression.

The ectopic cell cycle events and incomplete DNA replication in adult neurons may cause genomic instability and trigger sensors of DNA damage such as p53. To test this hypothesis, we first tested whether the overexpression of PCNA and DNA pol-β can induce the activation of p53 pathway. We found that neither PCNA overexpression nor DNA pol-β overexpression affected the protein level of p53 or the apoptotic rate. However, when the two proteins were overexpressed at the same time, the protein level of p53 increased significantly. Furthermore, the co-expression of PCNA and DNA pol-β also induced neuronal death, which was significantly attenuated by infection with p53 shRNA lentivirus (Figure 5A, B). We also investigated the role of p53 in MPP^+^-treated neurons. We found that the protein level of p53 increased significantly after MPP^+^ treatment, and the neuronal death induced by MPP^+^ was also attenuated by knocking down p53 (Figure 5C, D). These results indicate that the neuronal death mediated by the interaction of PCNA and DNA pol-β is p53-dependent.

It was reported that mutation of residues in the PCNA interaction motif (PIM)-like sequence II of DNA pol-β abolishes its binding to PCNA. We infected cultured neurons with retrovirus encoding PCNA together with retrovirus encoding wild-type DNA pol-β or H222A/F223A mutant DNA pol-β. Indeed, the mutant DNA pol-β was not co-immunoprecipitated by with PCNA, indicating the PIM-like sequence of DNA pol-β is required for its binding to PCNA (Figure 5E). We also detected the apoptotic rate by TUNEL staining. Interestingly, neuronal death induced by the overexpression of PCNA and DNA pol-β was significantly attenuated by DNA pol-β H222A/F223A mutation, indicating the interaction between PCNA and DNA pol-β is required for MPP^+^-induced neuronal death (Figure 5F, G).

## Discussion

In the present study, we provide evidence demonstrating that PCNA binds DNA pol-β, mediates ectopic DNA replication and neuronal death in an *in vitro* PD model. The DNA replication mediated by PCNA-DNA pol-β complex may be the missing link between the ectopic cell cycle events and neuronal death in PD and other neurodegenerative diseases.

The cell cycle is a series of events that take place in a cell leading to the generation of two daughter cells. In eukaryotes, the normal cell cycle consists of four phases: G1, S, G2, and M phase. During S phase, the DNA replication occurs. During M phase, the cell splits itself into two daughter cells. During G1 and G2 phase, the cells prepare for S phase and M phase, respectively. The mature neurons in the brain are terminally differentiated and exit the cell cycle. However, it has been reported that the vulnerable neurons re-entered the cell cycle in PD and AD brain. Some neurons even completed nearly full DNA replication of S phase [1], [4], [5], [23]. It is well-recognized that the unscheduled and incomplete cell cycle events mediate neurodegeneration [5], [24]. However, the molecular mechanisms of neuronal DNA replication are not clear.

We and others found that inhibition of the cell cycle progression or inhibition of DNA replication may attenuate neuronal death induced by various neurotoxins, indicating DNA replication in S phase plays a key role in neuronal death [10], [12], [25], [26]. We found that postmitotic neurons activated a non-canonical molecular mechanism of DNA replication after they were exposed to MPP^+^. During the S phase of a normal cell cycle, DNA pol-δ and DNA pol-δ mediate DNA replication. However, DNA pol-β was responsible for the neuronal DNA replication in MPP^+^-treated neurons. Furthermore, inhibition of DNA pol-β was protective against MPP^+^ toxicity [10]. DNA pol-β was also found to be the predominant DNA polymerase in neurons treated with amyloid β [14], [15]. These results suggest that DNA pol-β is required for neuronal death in neurodegenerative diseases. However, as shown in figure 4, the expression of DNA pol-β itself was not sufficient to induce neuronal death.

PCNA is well known as a processivity factor for DNA pol-δ. Once it is loaded into the DNA, it will stabilize the interaction with template DNA and slides along it stably. By doing so, it plays crucial rules in many aspects of DNA metabolism by mediating interactions of proteins with DNA. It is involved in normal DNA replication and several forms of DNA repair [19], [27], [28]. PCNA is not detected in adult neurons, but it was found in the nucleus of vulnerable neurons from PD patients as well as in the nigral dopaminergic neurons of 1-methyl-4-phenyl-1,2,3,6-tetrahydropyridine (MPTP)-treated mice [1]. Since we found that both PCNA and DNA pol-β are required for MPP^+^-treated neuronal death, we tested whether they bind to each other. The interaction of PCNA and DNA pol-β was confirmed by reciprocal co-immunoprecipitation. GST pull-down assay showed that PCNA binds to the catalytic domain of DNA pol-β. Interestingly, the binding of PCNA and DNA pol-β were also found in brain samples from PD patients. It has been reported previously that DNA pol-β contains sequences resembling the PCNA interacting motif. By using biochemical approaches such as co-immunoprecipitation, yeast two-hybrid analysis, and overlay binding assay, Kedar et al. demonstrated that the PCNA interacting motif sequence is required for its interaction with PCNA [19]. In the present study, we explored the pathological roles of the PCNA-DNA pol-β complex, and provide evidence that the interaction between PCNA and DNA pol-β is required for neuronal death induced by MPP^+^, because the mutant DNA pol-β that dose not binds to PCNA lost its neurotoxic effect (Figure 5F).

We also found that the PCNA and DNA pol-β coimmunoprecipitated with DNA primase and Cdc45, which were found exclusively at replication origins [20], [21], [29], indicting they are loaded into the DNA replication forks. More importantly, neither PCNA nor DNA pol-β was sufficient to initiate DNA replication. Increased DNA replication was detected only when the two proteins were expressed at the same time, indicating the interaction between them is required for neuronal DNA replication. How the ectopic DNA replication mediated by PCNA and DNA pol-β induces neuronal death is not fully understood, but it has been reported that DNA pol-β dose not have proofreading activity, and replicates DNA with low fidelity [30]. It is possible that DNA pol-β-mediated DNA replication will increase the mutagenesis rate and cause genomic instability [31]. The cells will active the sensors of DNA damage such as p53 [15], [32]. Indeed, we found that the overexpression of PCNA and DNA pol-β caused the overexpression of p53, and knockdown of p53 abolished neuronal death. Our results demonstrate that neuronal death induced by PCNA and DNA pol-β is p53-dependent.

In summary, this study demonstrates the interaction of PCNA and DNA pol-β causes “DNA replication stress” and contributes to neuronal death induced by MPP^+^. The aberrant DNA replication may represent the missing link between cell cycle events and neuronal death in PD.
